# Aquaporin AtTIP5;1 negatively regulates nitric oxide-promoted lateral root development by affecting auxin transport and homeostasis of reactive oxygen species in roots of *Arabidopsis*

**DOI:** 10.1080/15592324.2026.2739433

**Published:** 2026-09-26

**Authors:** Qihang Zhu, Panpan Li, Lirong Sun, Shibin He, Fushun Hao

**Affiliations:** a National Key Laboratory of Cotton Bio-breeding and Integrated Utilization, School of Life Sciences, Henan University, Kaifeng, China

**Keywords:** AtTIP5;1, lateral root, nitric oxide, auxin transport, reactive oxygen species, antioxidase

## Abstract

Aquaporins and nitric oxide (NO) are essential regulators of lateral root (LR) development in plants. However, whether an aquaporin could modulate NO-affected LR formation remains elusive. Here, we show that the tonoplast intrinsic aquaporin protein AtTIP5;1 negatively regulates NO-promoted LR development. AtTIP5;1 is highly expressed in root pericycle cells. *AtTIP5;1* overexpression causes significant suppression of LR formation induced by sodium nitroprusside (SNP), an NO donor. Moreover, *AtTIP5;1* overexpression results in a clear decrease in NO accumulation in the roots. The application of exogenous auxin influx inhibitor naphthoxyacetic acid boosts the effect of AtTIP5;1 on LR growth evoked by SNP, whereas the auxin efflux inhibitors N-1-naphthylphthalamic acid and 2,3,5-triiodobenzoic acid notably attenuate this effect. In addition, *AtTIP5;1* overexpression leads to elevated hydrogen peroxide levels, decreased superoxide anion accumulation, enhanced superoxide dismutase activity, and reduced activities of catalase, ascorbate peroxidase, and peroxidase in roots under SNP treatment. These results suggest that AtTIP5;1 may inhibit NO-facilitated LR development by reducing NO accumulation, affecting auxin transport, and altering ROS homeostasis in *Arabidopsis*.

## Introduction

Lateral roots (LRs) are vital for plant growth and survival by facilitating anchoring and the acquisition of water and nutrients from the soil. LRs originate from some pericycle founder cells positioned in the xylem pole in primary roots. The founder cells divide asymmetrically to form lateral root primordia (LRP), which further develop into LRs in plants.^1^ ^,^ ^2^ The formation of LRP can be divided into four stages by Zhang et al.^3^ or eight stages by Malamy and Benfey.^4^ The former are stage 1 (S1: 0–3 cell layers of LRP), stage 2 (S2: more than 3 cell layers of LRP but no LRs have yet appeared), stage 3 (S3: LRs length < 0.5 mm), and stage 4 (S4: LRs length > 0.5 mm).

LR development is controlled by many factors, for example, various phytohormones, including auxin, and different environmental cues, including water and nutrient availability. Among these, auxin plays a dominant role at all developmental stages of LR formation. Auxin activity, distribution, polar transport, and signaling in roots dramatically affect LR branching.^1^ ^,^ ^2^

LR formation is also regulated by some secondary messengers, such as reactive oxygen species (ROS) and nitric oxide (NO).^5^ ^,^ ^6^ ROS, including superoxide anion (⋅O_2_^–^) and hydrogen peroxide (H_2_O_2_), have been demonstrated to affect the induction and development of LRs. Many redox regulators are involved in these processes, for instance, transcriptional factor UPBEAT1 (UPB1), multiple peroxidases, and NADPH oxidase subunits.^5^ ^,^ ^7^ ^,^ ^8^ NO, which mainly works by causing S-nitrosylation of many proteins, has been suggested to positively regulate the initiation and elongation of LRs under normal conditions and in response to different environmental stresses.^6^ ^,^ ^9^ ^,^ ^10^ For example, NO promotes LR growth in tomato.^11^ It also facilitates LR formation in tomato and *Arabidopsis* after treatment with methane or in sunflower under salt stress.^12^ ^,^ ^13^

Aquaporins, including the plasma membrane intrinsic proteins (PIPs) and tonoplast intrinsic proteins (TIPs), are channel proteins located in cell membranes that mediate the transport of water and other small solutes across the membranes.^14^ Evidence indicates that aquaporins play essential roles in LR development by modulating water flow in roots. Péret et al.^15^ reported that auxin stimulates LR development by reducing *AtPIP2;1* expression in cortical cells but upregulating *AtPIP2;8* expression at the base of the LRP and underlying stele in *Arabidopsis*. Moreover, auxin-tightly-controlled water transport mediated by AtPIP2;1 is crucial for LR formation. In another study, AtPIP1;1 is described to inhibit LR development under low nitrate conditions.^16^ Additionally, Reinhardt et al.^17^ found that AtTIP1;1, AtTIP1;2, and AtTIP2;1 positively affect LR branching in *Arabidopsis*.

Both aquaporins and NO play essential roles in LR development. However, it is unknown whether an aquaporin plays a role in NO-modulated LR formation. Previously, we demonstrated that AtTIP5;1, an *Arabidopsis* tonoplast aquaporin, positively regulates hypocotyl elongation through mediating the gibberellin (GA) response under high-boron stress.^18^ Considering that some AtTIPs are important modulators of LR branching,^17^ we hypothesized that AtTIP5;1 may have similar roles as other AtTIPs in LR growth. To test this hypothesis, the possible roles of AtTIP5;1 in LR formation were investigated using its mutants and overexpression lines under different abiotic stresses, including high salinity and drought, as well as after treatment with the generators of ROS and NO. Interestingly, we found that AtTIP5;1 negatively regulated NO-promoted LR development. Moreover, auxin transport, ROS balance, and the activities of several antioxidant enzymes in roots were markedly affected by *AtTIP5;1* overexpression.

## Materials and methods

*Arabidopsis thaliana* wild type (WT) (Col-0), the homozygous T-DNA insertion mutants *tip5;1-1* (*m1*) and *tip5;1-2* (*m2*), as well as the *AtTIP5;1* overexpression lines OE4, OE9, OE14, and OE15^18^ were used in this study. The seeds of all genotypes above were sterilized with 0.1% sodium hypochlorite for 15 min, rinsed 8–10 times with sterile double-distilled water, and sown on MS solid medium (0.6% agar). After vernalization at 4 °C for 2 d, the seeds were cultured in a growth chamber under conditions of 16 h light and 8 h dark, 18–22 °C, and a light intensity of 120–150 μmol·m^−2^·s^−1^.

### Analyses of root phenotypes and LR density

Five-day-old seedlings with a primary root length of approximately 1 cm were transferred to MS (Murashige and Skoog) medium (1.0% agar) alone or MS medium containing different concentrations of sodium nitroprusside (SNP) (10, 20, and 30 μM) and 1-naphthaleneacetic acid (NAA) (0.03, 0.05, and 0.08 μM), and 100 μM 2-(4-carboxyphenyl)-4,4,5,5-tetramethylimidazoline-1-oxyl-3-oxide (c-PTIO), 1 μM 1-naphthoxyacetic acid (NOA), 1 μM naphthylphthalamic acid (NPA), or 2 μM 2,3,5-triiodobenzoic acid (TIBA) for another 12 d. The root phenotypes were observed, and the number of LRs that broke through the epidermis was counted under an Olympus stereomicroscope (CX41). The primary root length was measured with a ruler. The LR density (the number of LRs per centimeter of primary roots) was then calculated. The concentrations of the reagents above were selected based on previous reports,^19-21^ and our preliminary experiments.

### Analysis of *AtTIP5;1* expression signal

A DNA sequence harboring the promoter (1955 bp) and genomic DNA (834 bp) of *AtTIP5;1* gene was amplified by polymerase chain reaction (PCR) using two primers (5′-ATCTCATTGCCCCCCCGTTGATAGTCCAAAACGCACG -3′ and 5′-GTTCATTTCATTTGGAGAGGACAGCACACCAATGGCATCACC-3′). The obtained fragments were cloned into the vector *pCAMBIA1300:GFP* (replacing the *35S* sequence) and sequenced. A *Pro*_*AtTIP5;1*_ *:AtTIP5;1-GFP* construct was generated. Transgenic *Arabidopsis* plants expressing *Pro*_*AtTIP5;1*_:*AtTIP5;1-GFP* were created by the *Agrobacterium*-mediated floral dip method. *GFP* signal in the roots of 10-day-old *Pro*_*AtTIP5;1*_ *:AtTIP5;1-GFP* seedlings grown in MS medium was observed under a laser scanning confocal microscope (ZEISS LSM 700).

### Observation of lateral root primordia

Five-day-old seedlings were transferred to MS medium or MS medium supplemented with 10 μM SNP for another 1, 3, or 5 d. Seedlings were sampled. The number of LRP at four developmental stages (S1, S2, S3, and S4) was counted according to the method of Zhang et al.^3^, and the average number of LRP at each stage per seedling was calculated.

### NO detection

Five-day-old seedlings were transferred to MS medium containing 20 μM SNP for another 3 d. Then, the seedlings were immersed in 15 μM 4-amino-5-methylamino-2',7'-difluorofluorescein diacetate (DAF-FM DA) probe solution (5 mM 2-morpholinoethanesulfonic acid-KOH, pH 5.7, 0.25 mM KCl, 1 mM CaCl₂) and incubated for 45 min. After rinsing three times with the probe solution, the seedlings were mounted on slides, and the fluorescence in the mature root region of each seedling was observed using a laser scanning confocal microscope (ZEISS LSM 700) at an excitation wavelength of 488 nm and an emission wavelength of 500–550 nm in single-channel mode. Relative fluorescent intensity was the total fluorescence value of the mature root region divided by the total area of the region. Data are shown as the mean of at least 10 roots from three independent experiments.

### H_2_O_2_ and ⋅O_2_^–^ assays

Five-day-old seedlings were transferred to MS medium containing 20 μM SNP for 3 d. The seedlings with consistent growth status were immersed in 50 μM 2',7'-dichlorofluorescin diacetate (H₂DCFDA) or 30 μM dihydroethidium (DHE) working solution and incubated at room temperature for 15 min. After rinsing with solution buffer, the seedlings were mounted on slides and scanned with a laser scanning confocal microscope (ZEISS LSM 700) at an excitation wavelength of 488 nm and an emission wavelength of 500–550 nm for H₂DCFDA or an excitation wavelength of 561 nm and an emission wavelength of 570–620 nm for DHE in single-channel mode. Relative fluorescent intensities of H_2_O_2_ and ⋅O_2_^–^ in the root tip and the mature region were determined as the total fluorescence value divided by the total area in the root region. Data are shown as the mean of at least 10 roots of three independent experiments.

### Antioxidase activity analysis

Five-day-old seedlings were transferred to MS medium or MS medium containing 20 μM SNP for another 3 d. Roots (0.01 g) were sampled and used. The activities of superoxide dismutase (SOD), catalase (CAT), and peroxidase (POD) were measured according to the method as described previously.^22^ APX activity was detected using a CheKine™ Ascorbate Peroxidase micro‑volume assay kit (BORG Biotechnology Company, China) following its manufacturer’s protocols.

## Results

### *AtTIP5;1* overexpression causes the suppression of NO-promoted LR formation

To determine whether AtTIP5;1 exerts effects in NO-mediated LR development in *Arabidopsis*, we compared the differences in LR density among WT, *AtTIP5;1* mutants (*m1*, *m2*), and *AtTIP5;1* overexpression lines (OE4, OE9, OE14, and OE15) after treatment with SNP, a widely used NO donor. In the absence of SNP, no significant differences in LR density were observed among all the genotypes. In the presence of 10, 20, or 30 μM SNP, LR formation was clearly promoted in all the seedlings in a dose-dependent manner. Notably, the LR density of the four *AtTIP5;1* overexpression lines was markedly lower than that in WT, *m1*, and *m2* plants under all SNP treatments. No significant differences in LR density were found among WT, *m1*, and *m2* seedlings under the same treatment conditions. We further investigated the effect of NO scavenger c-PTIO on LR development evoked by 10 μM SNP. As expected, exogenous c-PTIO completely reversed the promotion effects of SNP on LR formation of all seedlings, especially the WT, *m1*, and *m2* lines (Figure 1). These results suggest that AtTIP5;1 negatively modulates NO-induced LR growth.

**Figure 1.**
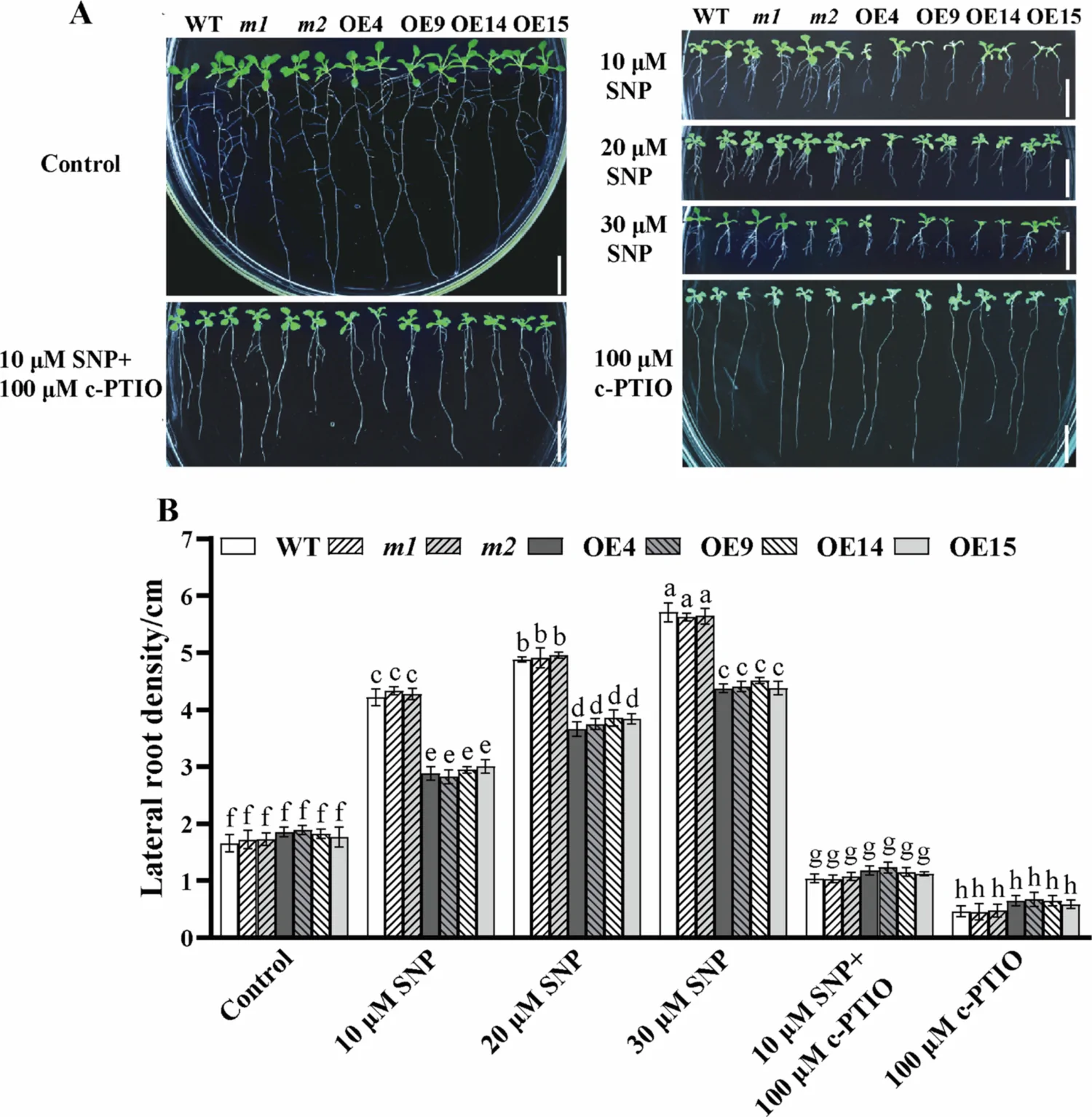
Effects of SNP and c-PTIO on LR formation of WT, *m1*, *m2*, OE4, OE9, OE14, and OE15 lines. (A) LR performances of various seedlings grown on MS medium containing different concentrations of SNP and/or c-PTIO. Scale bar = 1 cm. (B) LR density of WT, and *AtTIP5;1* mutants and overexpression lines. Data are means ± SD, *n* ≥ 30. Different lowercase letters denote significant differences between two lines by one-way ANOVA and Tukey’s HSD test (*P* < 0.05).

### AtTIP5;1 is active in the root pericycle and during LR development

We investigated the expression of *AtTIP5;1* in roots by generating transgenic *Arabidopsis* lines expressing a GFP reporter gene driven by the native promoter of *AtTIP5;1*. Clear GFP fluorescence signals were observed in the pericycle of primary roots and LRs (Figure 2), indicating that AtTIP5;1 may be an important regulator of LR branching in *Arabidopsis*.

**Figure 2.**
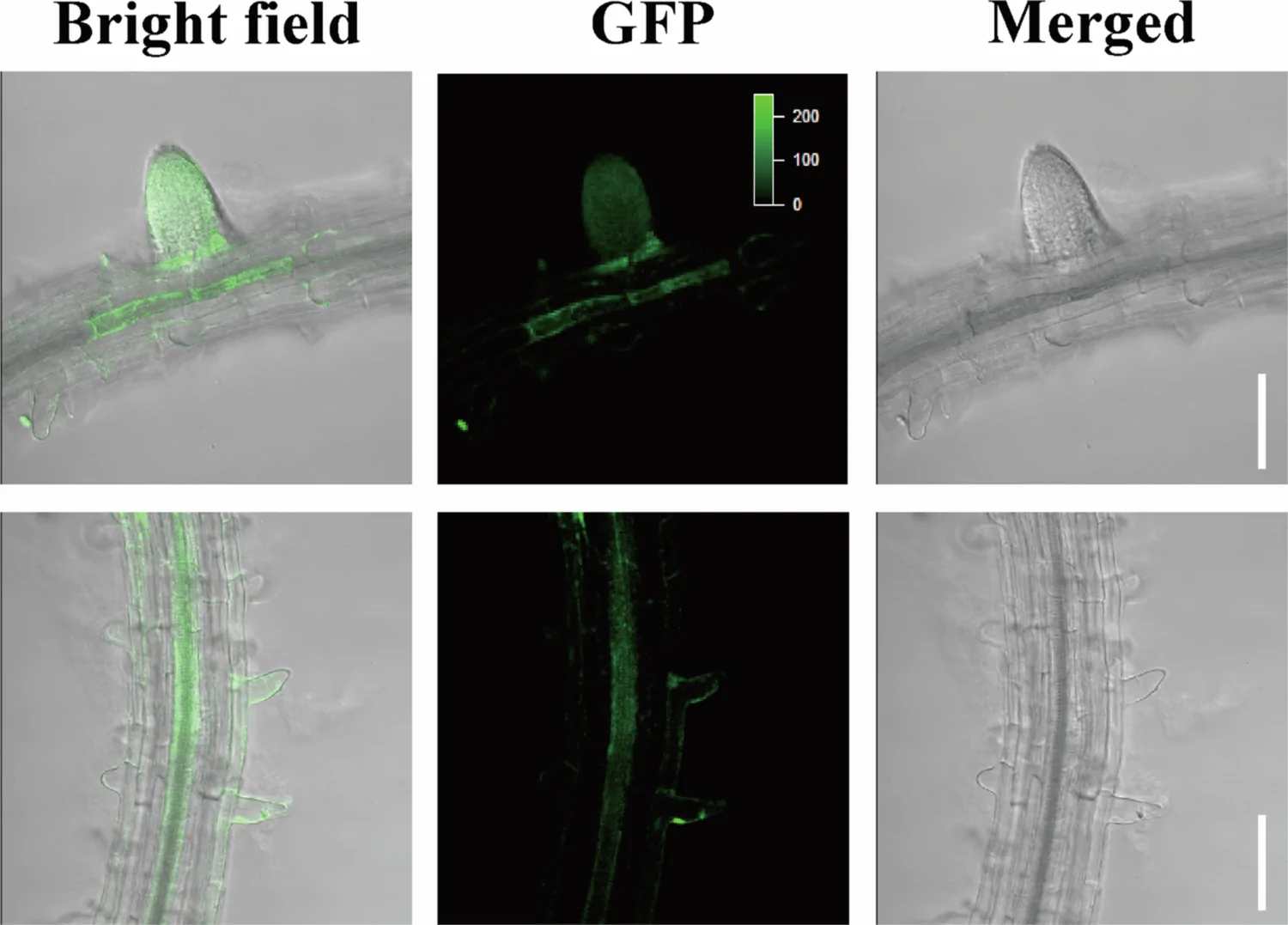
Expression pattern of *AtTIP5;1* in roots. Upper row, GFP fluorescence in the developing LR. Lower row, GFP fluorescence in the primary root. Ten-day-old seedlings grown in MS medium were observed. Scale bar = 100 μm.

### SNP-induced LRP emergence was delayed in *AtTIP5;1* overexpression lines

To define the role of AtTIP5;1 in LR development, LRP per plant was calculated in WT, *m1*, and OE9 after SNP treatment. After exposure of 5-day-old seedlings to 10 μM SNP for 1 d, the number of LRP at each developmental stage of all genotypes was similar (Figure 3A, B). Three days later, OE9 showed significantly fewer LRP than WT, and *m1* in the presence of SNP, with the most pronounced differences at stages S3 and S4 (Figure 3C, D). At day 5, most of the LRP in the WT and *m1* seedlings had progressed to stage S4. In contrast, very few LRP were detected at stage S4 in the overexpression line (Figure 3E, F). These results indicate that *AtTIP5;1* overexpression results in the inhibition of NO-promoted LRP initiation and progression.

**Figure 3.**
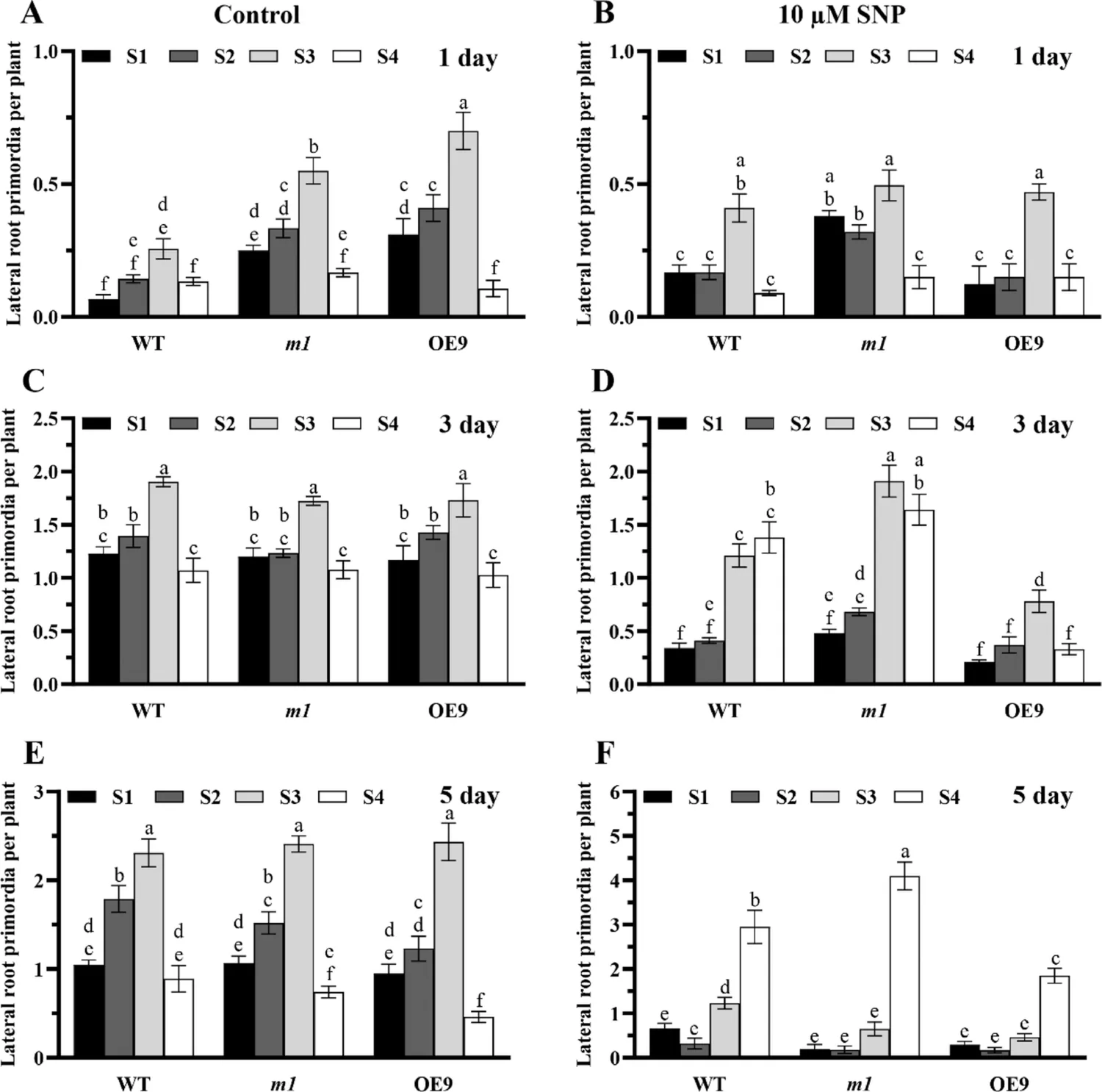
Development of LRP in WT, *m1*, and OE9. (A–C) LRP formation in different seedlings. LRP were observed after transferring 5-day-old seedlings to MS medium or SNP-containing medium for another 1, 3, and 5 d. Data are means ± SD, *n* ≥ 45. Different lowercase letters reveal significant differences between two seedlings by one-way ANOVA and Tukey’s HSD test (*P* < 0.05).

### NO accumulation is reduced in roots of *AtTIP5;1*-overexpressing seedlings after SNP treatment

To test whether AtTIP5;1 modulates LR development by changing the intracellular NO content, NO levels in the roots of WT, *m1*, OE9, and OE15 after SNP treatment were measured using DAF-FM DA, a NO-specific fluorescent probe. The results showed that NO accumulation in the roots of OE9 and OE15 was remarkably less than that of WT and *m1* (Figure 4). These results suggest that *AtTIP5;1* negatively regulates endogenous NO production during LR formation in response to SNP treatment.

**Figure 4.**
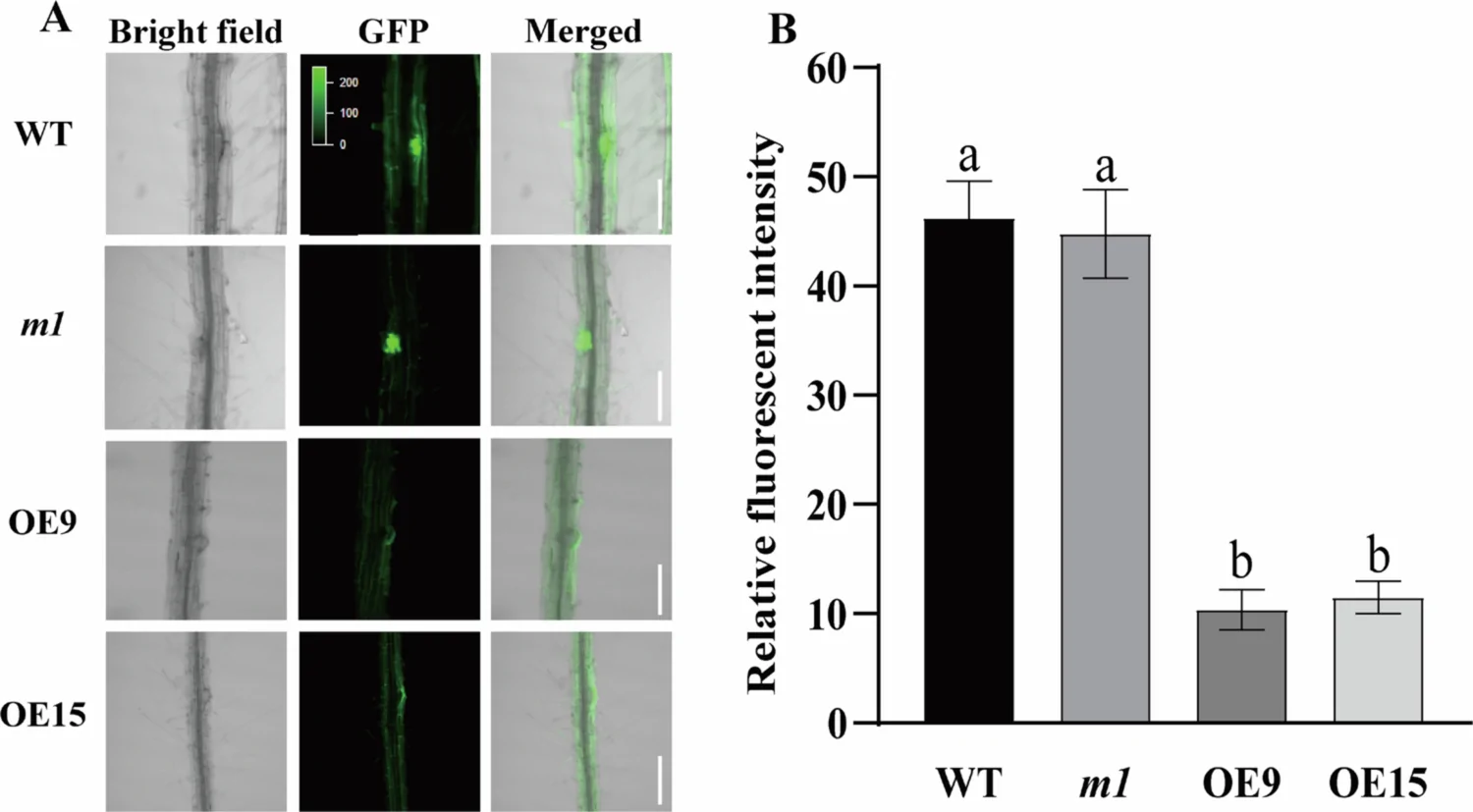
NO distribution in the roots of WT, *m1*, OE9, and OE15 seedlings. (A) Images of NO fluorescence in the roots of WT, *m1*, OE9, and OE15 seedlings after treatment with 20 μM SNP. Scale bar =100 μm. (B) Relative NO fluorescence intensity of the indicated genotypes in (A) (*n*≥ 16). Different lowercase letters indicate significant differences between two lines by one-way ANOVA and Tukey’s HSD test (*P*< 0.05).

### The inhibitory effects of AtTIP5;1 on SNP-stimulated LR development are boosted by NOA but attenuated by NPA and TIBA

Auxin plays key roles in modulating NO-mediated LR development. To explore whether AtTIP5;1-regulated LR branching is associated with auxin signaling, the effects of exogenous auxin (NAA) were tested. Treatment with 10 μM SNP evidently promoted LR formation in the WT, *AtTIP5;1* mutants, and overexpression lines. The application of NAA markedly increased LR density of all the seedlings in the presence of SNP, and the increased effects on all the genotypes were similar (Figure 5A, B), suggesting that auxin is not involved in AtTIP5;1-mediated LR development in response to SNP. To determine whether auxin transporters play roles in LR development regulated by NO, as well as AtTIP5;1, WT and *AtTIP5;1* mutants and overexpression lines were treated with the auxin influx inhibitor NOA, or the efflux inhibitors NPA and TIBA. The application of 1 μM NOA clearly enhanced the LR density of all the genotypes regardless of the SNP treatment. Moreover, the increased effects of NOA on LR formation of the four *AtTIP5;1* overexpression lines were prominently less than other genotypes, especially with SNP treatment. Either application of exogenous NPA or TIBA noticeably decreased the LR density of all the genotypes regardless of SNP treatment. Notably, the decreased effects of NPA or TIBA on LR development of *AtTIP5;1* overexpression lines were remarkably less than those of WT and *AtTIP5;1* mutants only in the presence of SNP (Figure 5). These results suggest that AtTIP5;1 may regulate NO-promoted LR development by inhibiting both influx and efflux of auxin in roots.

**Figure 5.**
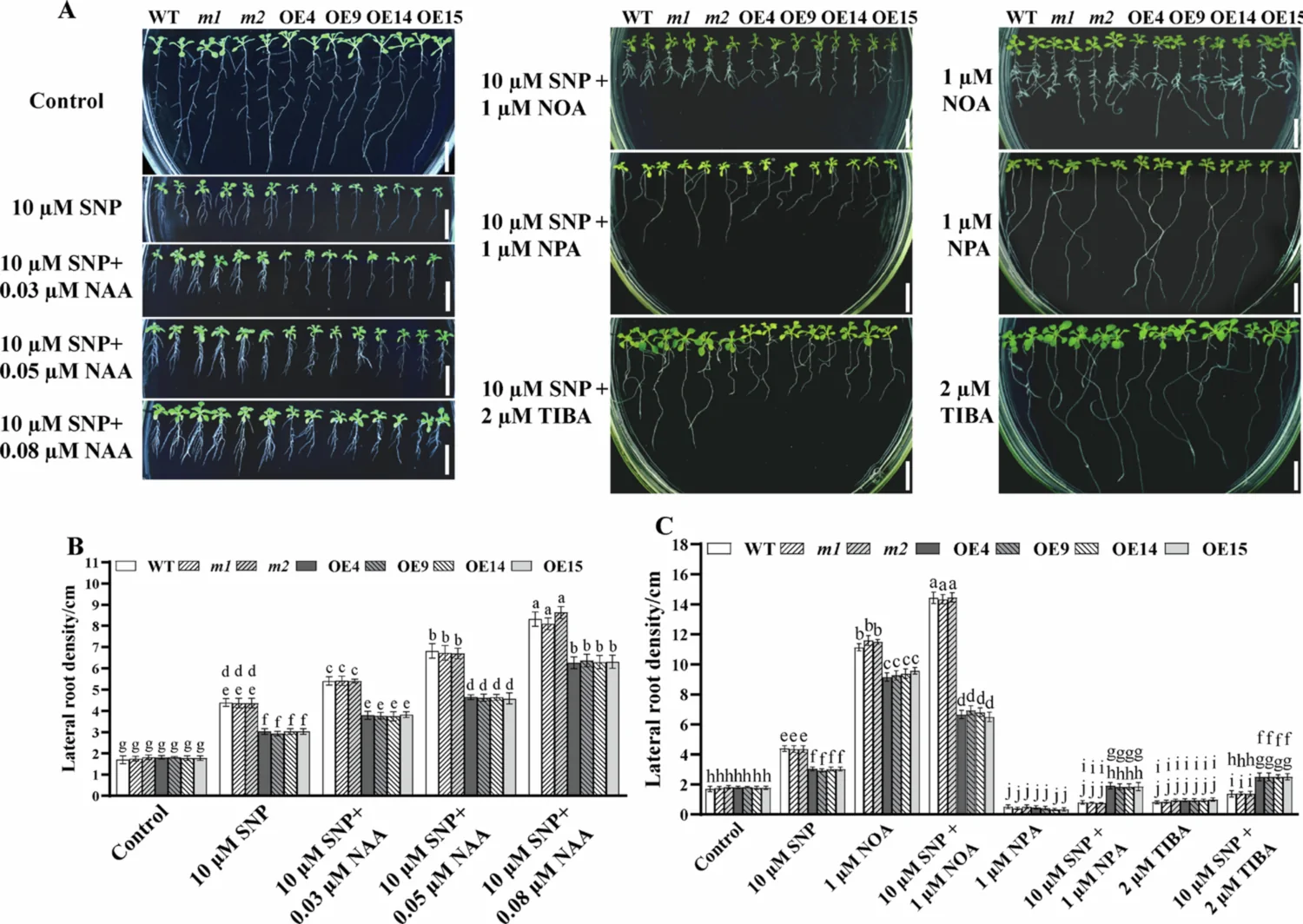
Effects of NAA, NOA, NPA, and TIBA on LR development under SNP treatment. (A) LR phenotypes of different seedlings after treatment with different concentrations of NAA, NOA, NPA, and TIBA combined with or without SNP. Scale bar = 1 cm. (B, C) LR density in (A). Data are means ± SD, *n* ≥ 30. Different lowercase letters show significant differences between two seedlings by one-way ANOVA and Tukey’s HSD test (*P* < 0.05).

### *AtTIP5;1* overexpression promotes H₂O₂ accumulation but decreases ⋅O_2_^–^ generation in roots in response to SNP treatment

ROS are key regulators of LR development. To determine whether AtTIP5;1 plays a role in NO-modulated LR formation through affecting ROS production, the levels of H₂O₂ and ⋅O_2_^–^ in the mature root zone and root tips of seedlings were examined using fluorescent probes H2DCFDA and DHE, respectively. In the absence of SNP, the fluorescence intensities of H₂DCFDA in the roots of WT, *m1*, OE9, and OE15 plants were similar. SNP clearly decreased H₂O₂ accumulation in the roots of all the genotypes. It is worth noting that the decreased effects of SNP in OE9 and OE15 were clearly less than those in WT and *m1* (Figure 6A–C). DHE assays showed that WT, *m1*, OE9, and OE15 had similar fluorescent signals without SNP treatment. The application of SNP noticeably reduced the fluorescence intensities of all the seedlings. Unexpectedly, the reduced effects of SNP on OE9 and OE15 but not *m1* were markedly higher than those of WT (Figure 6D–F). These findings indicate that AtTIP5;1 may regulate SNP-induced LR development by facilitating H₂O₂ generation and suppressing ⋅O_2_^–^ production in *Arabidopsis*.

**Figure 6.**
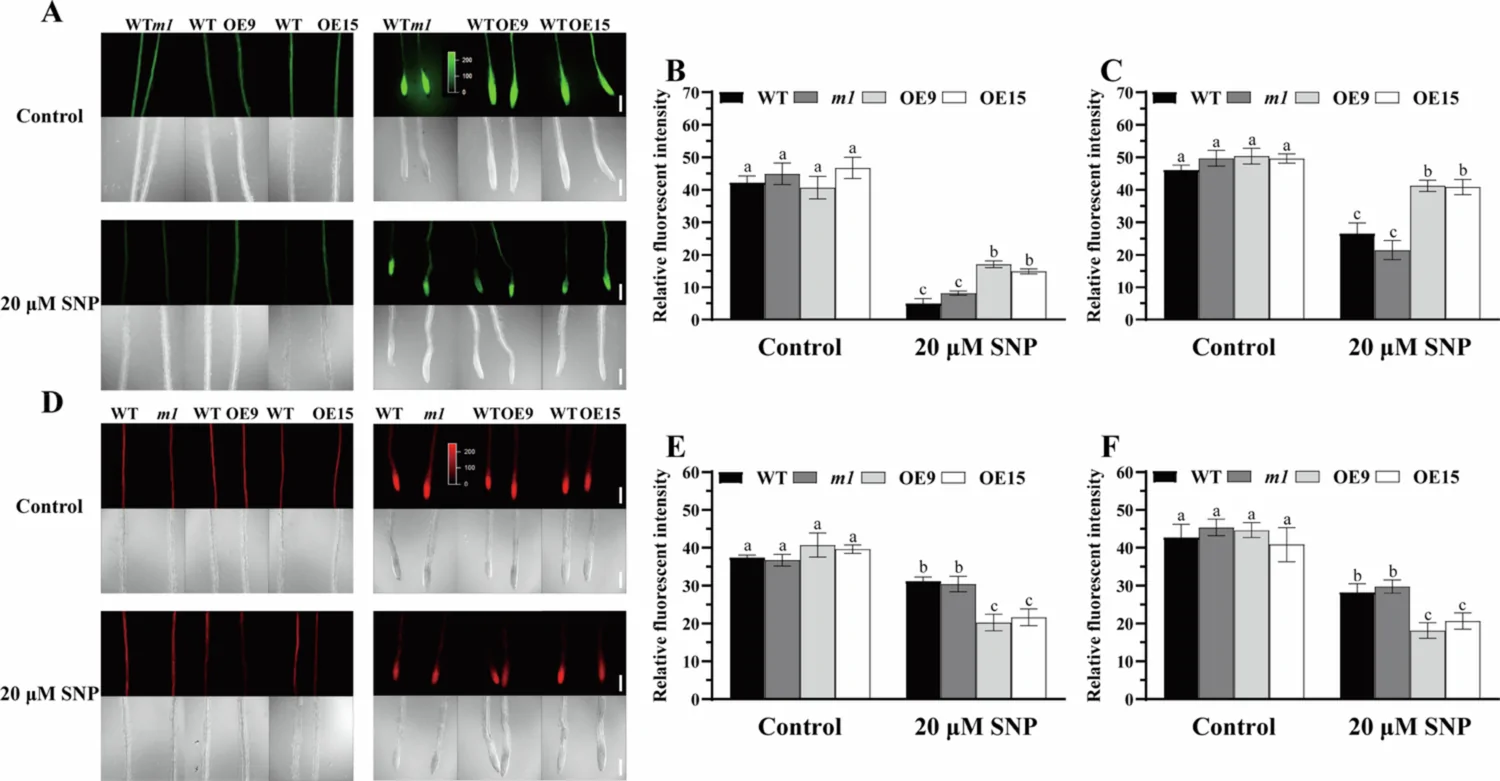
Levels of H_2_O_2_ and ⋅O_2_^–^ in seedling roots. (A) H_2_O_2_ generation was detected by H2DCFDA assays in the maturation zone and the root tip. (B, C) Relative fluorescent intensities in (A). (D) ⋅O_2_^–^ accumulation was visualized by DHE in the maturation zone and the root tip. (E, F) Relative fluorescent intensities of DHE in (D). Scale bar =100 μm. Data are the means ± SD, *n*≥ 16. Different lowercase letters reveal a significant difference between two seedlings by one-way ANOVA and Tukey’s HSD test (*P*< 0.05).

### The activities of SOD, CAT, APX, and POD are changed in *AtTIP5;1* overexpressors upon SNP treatment

It has been demonstrated that NO upregulates the activities of multiple antioxidases in plants, especially under stress.^23^ ^,^ ^24^ We therefore investigated the effects of the SNP on the activities of SOD, CAT, APX, and POD in the roots of WT, *m1*, OE9, and OE15. Treatment with 20 μM SNP significantly increased the activities of the four antioxidases in all the genotypes. However, the SOD activities in OE9 and OE15 were clearly higher than those in WT and *m1*, whereas the activities of the other antioxidases in the two overexpressors of *AtTIP5;1* were evidently lower than those in the WT and *m1* line (Figure 7). These results suggest that AtTIP5;1 has positive effects on NO-triggered SOD activity but has negative effects on the activities of CAT, APX, and POD stimulated by NO.

**Figure 7.**
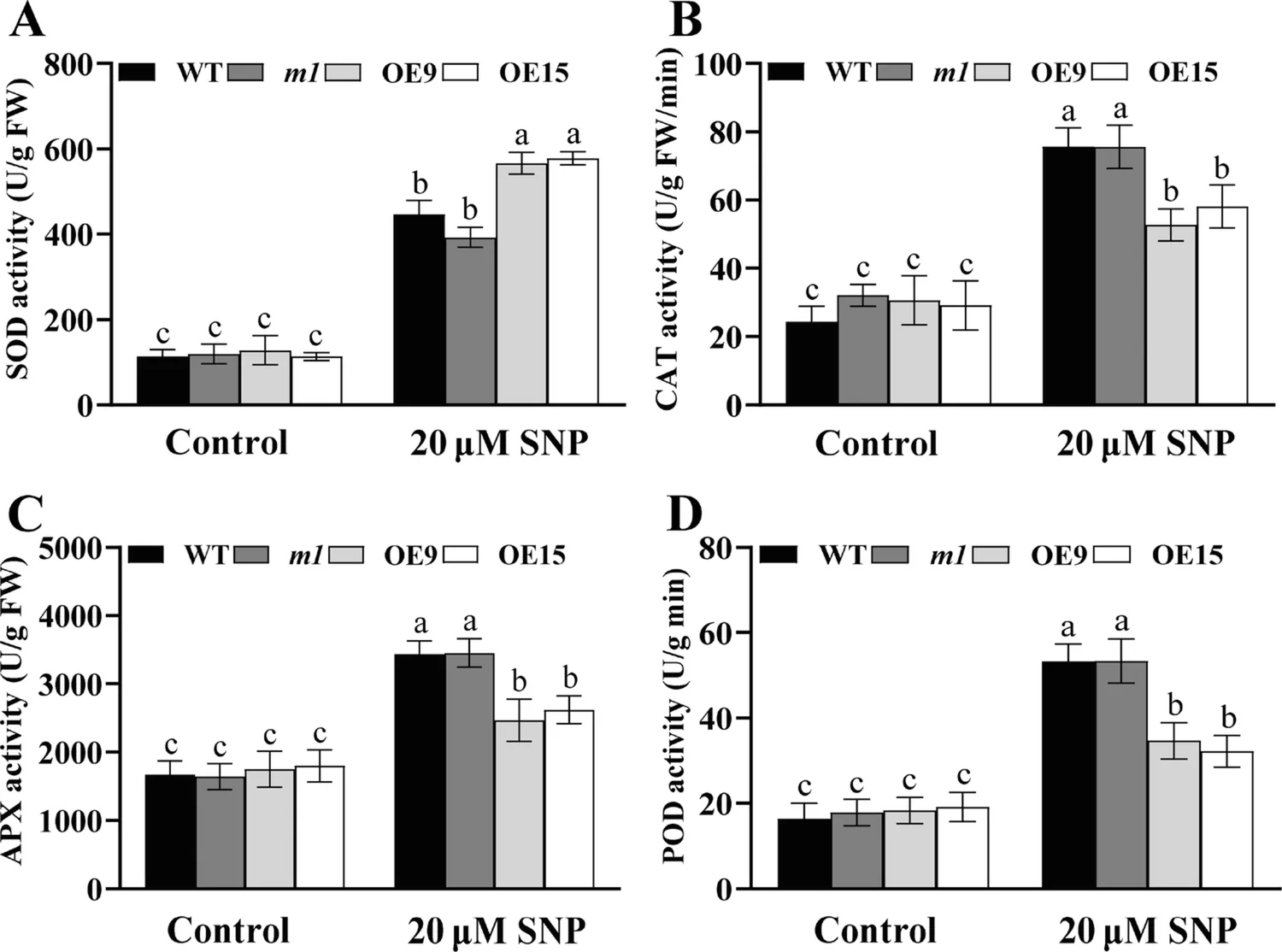
The activities of four antioxidases in the roots of WT, *m1*, OE9, and OE15 seedlings after treatment with 20 μM SNP. (A) SOD activity. (B) CAT activity. (C) APX activity. (D) POD activity. Data are means ± SD, *n*≥ 16. Different lowercase letters reveal a significant difference between two seedlings by one-way ANOVA and Tukey’s HSD test (*P*< 0.05).

## Discussion

Aquaporins have been implicated in modulating NO signaling during seed germination and in response to saline stress and hypoxia in plants.^25-27^ However, the exact roles of aquaporins in mediating NO signaling in plants have not been reported. In this study, we provide evidence that the *AtTIP5;1* gene was highly expressed in pericycle cells and LRs (Figure 2) and that its overexpression resulted in the inhibition of NO-stimulated LR development. AtTIP5;1 may exert effects through reducing NO levels, influencing auxin polar transport, and changing the accumulation of H_2_O_2_ and ⋅O_2_^–^ and the activities of several antioxidant enzymes in *Arabidopsis* roots.

TIPs are important regulators of LR branching in plants. *Arabidopsis* AtTIP1;1, AtTIP1;2, and AtTIP2;1 have been reported to promote LR development.^17^ However, we found that AtTIP5;1 had negative effects on NO-facilitated LR formation (Figure 1). These results suggest that TIPs can both positively and negatively affect LR formation, and that the underlying mechanisms may be complex.

We observed that *AtTIP5;1* overexpression led to a clear reduction of NO accumulation in roots after SNP treatment (Figure 4). The main reasons may be as follows. NO levels in cells and tissues are controlled by the equilibrium between NO production and elimination. NO is synthesized through multiple oxidative and reductive enzymatic reactions and nonenzymatic processes. It can be eliminated by reacting with oxygen, hydrogen sulfide, glutathione, many proteins, transcription factors, and other components, including ⋅O_2_^–^.^28-30^ In the presence of SNP, NO production is markedly enhanced. Consequently, cellular NO‑consuming reactions are promoted. TIPs are considered to control the transport of water and small solutes between the cytosol and the vacuole. They modulate vacuolar enlargement, cell elongation, and turgor maintenance of cells and tissues, all of which are critically required for numerous physiological and biochemical processes in plants.^14^ ^,^ ^17^ ^,^ ^31^ *AtTIP5;1* overexpression may lead to rapid exchange of water between the cytosol and the vacuole, markedly increasing cellular turgor and hydraulic conductance in tissues. These changes facilitate the movement of NO and accelerate NO degradation, thereby reducing NO accumulation in roots.

Much line evidence indicates that NO stimulates LR formation depending upon auxin or cooperating with auxin in plants. Moreover, NO is induced by auxin and is required for auxin-mediated LR development.^10^ ^,^ ^21^ ^,^ ^32^ Thus, NO can work in parallel with auxin or function both upstream and downstream of auxin in modulating LR branching. One key mechanism for NO regulating LR formation is that NO may change the activities of auxin transporters through S-nitrosylation.^10^ ^,^ ^32^ In this report, we showed that *AtTIP5;1* overexpression regulated NO-induced LR formation by inhibiting auxin efflux and influx in roots (Figure 5). The underlying mechanism may be complex. NO-mediated S-nitrosylation has been postulated to reduce the activities of the auxin efflux carriers PIN-FORMED 1 (PIN1) and PIN2 in *Arabidopsis*.^9^ Consistently, defects in protein de-nitrosylation caused by loss of function of S-nitrosoglutathione reductase 1 (GSNOR1) result in clearly increased cellular level of GSNO (a NO donor) and decreased activities of PIN1, PIN2, PIN3, PIN4, and PIN7.^33^ Under cadmium stress, increases in NO synthesis cause decreased accretion of PIN1, PIN3, and PIN7 in *Arabidopsis* roots.^34^ These data indicate that NO negatively modulates auxin efflux. *AtTIP5;1* overexpression may boost the inhibitory reactions of NO on auxin efflux through mediating rapid water flow and hydraulic adjustment, accordingly negatively modulating auxin efflux in SNP-stimulated LR growth.

There are very few reports on the effect of NO on auxin influx. Piacentini et al.^35^ found that SNP application enhances the intensities of expression signals of the rice auxin influx carrier gene *AUXIN1* (*AUX1*) in roots after treatment with cadmium, especially with arsenic. Likewise, Zhang et al. ^36^ described that SNP has enhanced effects on the expression of *AUX1* gene in cucumber roots in the presence of high nitrate and melatonin. These results imply that NO may positively impact auxin influx in plants under stress. The mechanism by which *AtTIP5;1* overexpression exerts inhibitory effects on NO-promoted auxin influx remains unknown. Most likely, the conditions of NO facilitating auxin influx are under abiotic stresses, which are significantly different from the conditions in our study.

ROS have been documented to act downstream of NO or collaborate with NO to regulate LR formation.^37^ In the present study, we observed that SNP application notably decreased the generation of both H_2_O_2_ and ⋅O_2_^–^ in roots. Intriguingly, the levels of H_2_O_2_ in two *AtTIP5;1* overexpressors were significantly higher than those in WT and *m1*, whereas the levels of ⋅O_2_^–^ in the two overexpressors were noticeably lower than those in the WT and *m1* strains (Figure 6). One possible reason for our findings is that NO directly reacts with and scavenges ⋅O_2_^–^ or inhibits ⋅O_2_^–^ generation through S-nitrosylation-mediated inactivation of NADPH oxidase.^23^ ^,^ ^32^ ^,^ ^37^ *AtTIP5;1* overexpression may promote these reactions by facilitating rapid water flux in cells. Accordingly, the ⋅O_2_^–^ levels in the two overexpressors of *AtTIP5;1* were low compared with those in the WT and *m1*. *AtTIP5;1* overexpression may also regulate ROS balance by affecting the activities of SOD, CAT, APX, and POD in the presence of SNP. Indeed, the SOD activities in the two *AtTIP5;1* overexpressors were notably higher than those in the WT and the *AtTIP5;1* mutant *m1*, whereas the activities of CAT, APX, and POD in the two overexpressors were markedly lower than those in the WT and *m1* line after SNP treatment (Figure 7). It is well known that SOD catalyzes the dismutation of ⋅O_2_^–^ into H_2_O_2_, whereas CAT, APX, and POD convert H_2_O_2_ into H_2_O. Changes in the activities of these antioxidases in *AtTIP5;1* overexpressors can contribute to high levels of H_2_O_2_ and low levels of ⋅O_2_^–^ in roots.

The molecular mechanism underlying the differential effects of high *AtTIP5;1* abundance on SOD, CAT, APX, and POD activities under SNP treatment remains unknown. It has been documented that the activities of antioxidant enzymes are modulated by NO-mediated S-nitrosylation and tyrosine nitration. NO also affects the expression of antioxidase genes and causes alterations in the expression of antioxidase genes by regulating numerous transcription factors and mitogen-activated protein kinases. Moreover, NO exerts diverse effects on different antioxidant enzymes.^23^ ^,^ ^24^ ^,^ ^30^ *AtTIP5;1* overexpression may accelerate these complex reactions or physiological processes by altering water transport and cellular turgor, further affecting the activities of SOD, CAT, APX, and POD. Alternatively, multiple TIPs have been reported to transport H_2_O_2_ in plants.^31^ ^,^ ^38^ *AtTIP5;1* may also facilitate H_2_O_2_ diffusion. Accordingly, rapid diffusion or compartition of H_2_O_2_ mediated by AtTIP5;1 in cells may differently modulate the activities of SOD, CAT, APX, and POD, further impacting the balance of ROS.

Based on our results, we propose a working model for the roles of AtTIP5;1 in *Arabidopsis* (Figure 8). In the presence of SNP, *AtTIP5;1* overexpression negatively regulates NO accumulation. Auxin transport mediated by influx carriers is attenuated, while auxin transport modulated by efflux carriers promotes SNP-induced LR branching. Elevated abundance of AtTIP5;1 suppresses both the auxin influx and efflux responses described above. *AtTIP5;1* overexpression also exerts positive effects on the suppression of NO on ⋅O_2_^–^ accumulation and has negative effects on NO-mediated inhibition of H_2_O_2_ production. In conclusion, AtTIP5;1 may repress NO-stimulated LR formation by decreasing NO levels, suppressing the roles of auxin transport carriers, and altering ROS balance in *Arabidopsis* roots. Our important work in the future is to determine the detailed roles and molecular mechanisms of AtTIP5;1 in NO signaling during LR development.

**Figure 8.**
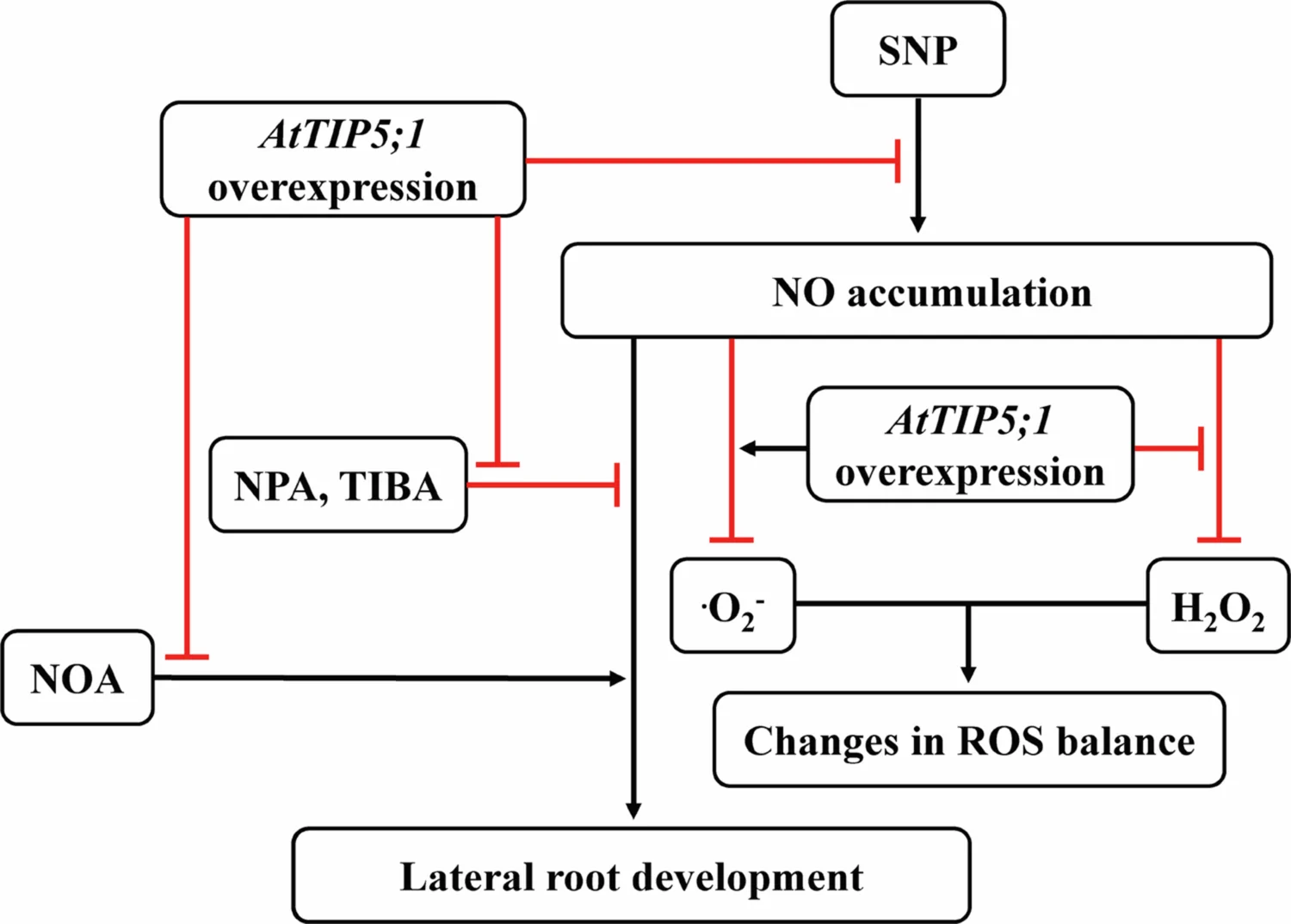
The working model of *AtTIP5;1* in NO-promoted LR formation. Elevated AtTIP5;1 abundance inhibits NO generation induced by SNP. The auxin influx inhibitor NOA stimulates, whereas the auxin efflux inhibitors NPA and TIBA suppress NO-facilitated LR formation. *AtTIP5;1* overexpression has negative roles on the effects of all inhibitors above. *AtTIP5;1* overexpression increases the suppression effect of NO on ⋅O_2_^–^ production and reduces the inhibitory effect of NO on H_2_O_2_ accumulation in the roots of *Arabidopsis*. The arrows indicate positive regulation, and the bars show negative regulation.
